# Development and Characterization of Active Pectin–Curdlan Biopolymer Films with Cannabigerol (CBG) Oil as Innovative Materials with Plant Metabolism–Stimulating Properties and Potential to Extend the Postharvest Shelf Life of Blackberries (*Black Satin*) Fruits

**DOI:** 10.3390/polym18070890

**Published:** 2026-04-06

**Authors:** Renata Dobrucka, Maja Paterska, Marcin Szymański

**Affiliations:** 1 Department of Non-Food Products Quality and Packaging Development, Institute of Quality Science, Poznan University of Economics and Business, al. Niepodległości 10, 61-875 Poznan, Poland; 2 Department of Plant Physiology, Poznan University of Life Sciences, Wołynska 35, 60-637 Poznan, Poland; 3 Center for Advanced Technologies, Adam Mickiewicz University in Poznan, ul. Uniwersytetu Poznańskiego 10, 61-614 Poznan, Poland

**Keywords:** cannabigerol, *Thomson seedless* dark grapes, natural preservative

## Abstract

In the present study, the physicochemical, mechanical, and functional properties of biodegradable pectin/cudlan gum polysaccharide films with CBG oil were evaluated. In these studies, the TS values for the films ranged from 8.50 MPa to 14.80 MPa. The EB values ranged from 33.06% to 39.07%. The WVTR ranged from 13.7 to 9.51 g/m^2^ d. In all the films tested, the change in the L* parameter did not change significantly statistically (*p* ≥ 0.05). In films with low CBG content (0.125F, 0.25F, 0.35F), L* remained stable, which indicated their resistance to darkening. However, film 0.5F was an exception, as it showed a decrease in L*, suggesting darkening or photodegradation processes. CBG films reduced mold growth, water loss, color degradation, and anthocyanin content in stored fruit, especially films with a content of 0.125F–0.35F, while higher concentrations (0.5F–0.75F) could cause pro-oxidative effects. Soil application of the film showed that moderate CBG concentrations (0.25F–0.35F) increased the content of chlorophyll, carotenoids, and phenols, indicating biostimulating potential, while the highest concentrations could cause oxidative stress. At the highest CBG concentration (0.75F), the carotenoid content decreased to 0.054–0.113 mg·g^−1^ FW. At higher concentrations of active substances in the film (0.5F and 0.75F), stabilization or a decrease in O_2_•^−^ levels was observed, which may indicate the effective activation of protective mechanisms leading to the neutralization of excess free radicals.

## 1. Introduction

Blackberries, especially the *Black Satin* variety, which is thornless and the earliest fruiting blackberry variety, are widely valued for their attractive sensory properties and high content of bioactive compounds. They grow to a height of 3 to 5 m, forming dense, compact clumps. The fruit is characterized by its sweet taste and high nutritional value, which is mainly due to its vitamin and mineral content [1]. They contain significant amounts of phenolic compounds, including anthocyanins, flavonols, chlorogenic acid, and procyanidins, which are characterized by high biological activity and may provide health benefits as antioxidants in the diet [2]. Blackberries are a valuable source of ellagitannins. Ellagitannins are esters of 3,4,5,3′,4′,5′-hexahydroxydiphenyl acid (HHDP acid) and polyhydric alcohol (monosaccharide), usually β-D-glucose (or its oligomers). It is believed that some of the health-promoting properties of ellagitannins are related to their ability to release free ellagic acid from their molecules and its further metabolism in humans and animals. The metabolism of ellagitannins by the bacterial flora of the gastrointestinal tract results in the prolonged release of ellagic acid into the blood [3]. Their delicate texture and high water activity make them highly perishable, leading to rapid microbial proliferation and deterioration in quality during storage. This poses a significant challenge in extending their shelf life while maintaining their nutritional and organoleptic qualities [4]. Nowadays, with the growth of the world’s population and the need to provide sufficient food for consumers around the world, maintaining the quality of fresh food products and extending their freshness is extremely important [5]. Their appropriate packaging will also play a huge role. The vast majority of food packaging on the market is made from synthetic materials derived from non-renewable sources. Despite having the right barrier and mechanical properties, they cause huge amounts of non-degradable solid waste to be released into the environment, causing environmental problems [6]. In response to rising environmental concerns and the demand for eco-friendly solutions, the use of biopolymer-based packaging has expanded rapidly as a substitute for traditional plastics [7]. A forthcoming regulation on packaging materials represents a crucial step toward reducing the environmental challenges posed by petrochemical-based alternatives [8,9]. Today, the European Union is undertaking a series of measures to fight plastic pollution and accelerate the transition to a circular plastics economy. Some proposed measures specifically target plastics and packaging waste, with the ultimate goal of achieving climate neutrality by 2050 [10,11].

Scientists have been conducting research for years to develop packaging materials that meet increasingly stringent requirements. The emergence of biopolymers has initiated this transformation, offering properties comparable to their fossil fuel-based counterparts, while coming from renewable sources. Biopolymer-based materials are very interesting from the point of view of sustainability and consumer safety [12,13,14,15]. The importance of these materials lies in their biodegradability, compostability, and renewability, which contributes to reducing the environmental footprint associated with packaging waste. They are expected to become price-competitive in the future, given the limited supply of fossil resources and advances in production technologies [16]. Researchers are investigating the possibility of producing biodegradable packaging from biomass materials such as polysaccharides and proteins [17]. Polysaccharides, one of the most abundant biopolymers in nature, are long-chain macromolecules formed by the combination of monosaccharides via glycosidic bonds [18]. In order to gain active functions, such as antibacterial or antioxidant properties, they often require the addition of additives to the polymer matrix [19,20,21]. These are most often plant extracts, nanofillers, or essential oils.

This study developed films based on selected polysaccharides: pectin and curdlan gum. These are polysaccharides with gelling and stabilizing properties, recognized as food additives by the WHO/FAO Expert Committee. Pectin (E440) is an inexpensive anionic polysaccharide used in the production of agricultural, agri-food, and food waste approved by the FAO and the EU, such as apple pomace, orange fiber, and plant cell walls [22]. On the other hand, pectin can also be extracted from plant cell walls by isolation, as the main component comes from (~35%) dicotyledonous and monocotyledonous plants other than grasses [23]. It consists of β-(1-4)-D-galacturonic acid linked by molecular bonds to galactose and rhamnose [24]. In the pectin structure, the carboxyl group of the sugar molecule can be partially esterified with a methyl group and partially or completely neutralized with one or more bases. The presence of electron-donating groups, such as carboxyl, hydroxyl, and methoxyl groups (especially in galacturonic acid), is responsible for the antioxidant activity of pectin [25]. Curdlan gum (E424) is a linear glucose polymer, an exopolysaccharide, specifically a homopolysaccharide, consisting exclusively of glucose monomers linked by β-1,3-glycosidic bonds [26]. It is a microbial extracellular polysaccharide (EPS), typically produced by *Agrobacterium* sp., *Rhizobium* sp., *A. faecalis*, *Gluconacetobacter xylinus*,* Bacillus cereus*, and other microorganisms. Bacteria usually produce curdlan in response to stress factors in the external environment [27]. It is a commonly used thickener and stabilizer in the food and pharmaceutical industries. Curdlan has anticancer, antibacterial, and antiviral properties. The intramolecular and intermolecular hydrogen bonds of curdlan provide excellent film-forming properties, making it a suitable component of biodegradable film matrices [28].

Pectin film in pure form has poor mechanical properties and high viscosity, which makes it unsuitable for packaging fruit and vegetables. However, pectin exhibits high hydrophilicity, and its combination with other substrates can significantly improve the properties of the film. In addition, pectin has become a carrier of antioxidants and antibacterial agents due to its biocompatibility, widespread availability, low cost, and low toxicity [29]. There are many examples in the world literature of the production of pectin-based packaging materials, as it is highly compatible with other biopolymers such as proteins, polysaccharides, and synthetic biopolymers. An example is the work of Bhatia et al. [30], who developed an edible film composed of pectin and xanthan gum with the addition of grapefruit essential oil. The results showed that the film had beneficial properties, increasing antioxidant properties. Biratu et al. [31] investigated the presence of honey and propolis in different concentrations of pectin. Pectin-based films with the addition of propolis showed better antioxidant properties than honey and inhibited the growth of Gram-positive bacteria. Pectin was also used as a matrix in the production of films using wine pomace extracts as biocomponents with antioxidant activity.

Additionally, citrus pectin and guar gum with the addition of *Rumex hydrolapathum* extract were used to develop active films against *E. coli* [32]. With an increase in the concentration of the extract in the tested film, an increase in the effectiveness of inhibiting the growth of this microorganism was observed. A combination of apple pectin and citrus pectin in a ratio of 80:20 was also combined with extract from unhusked *Cannabis sativa* L. seeds. Tests showed that the prepared films exhibited inhibitory activity against all tested microorganisms. All prepared films showed antibacterial activity against *Salmonella typhimurium* and *L. monocytogenes* strains [33]. Pectin films with antioxidant activity were also obtained with the addition of *Cannabis sativa* L. herb extract [14]. To date, there have been no reports on packaging materials with the addition of CBG oil. Cannabigerol (CBG) is a non-psychotropic cannabinoid obtained in 1964 by Gaoni and Mechoulam, which does not produce effects similar to Δ9-THC in vivo. To date, CBG has been shown to have antiproliferative, antibacterial, and anti-glaucoma effects, as well as antagonistic effects on the antiemetic action of CBD [34]. From an industrial perspective, the growing interest in CBG stems from its lack of psychoactive effects and its wide range of potential applications. Since CBG has a unique pharmacological profile and high translational potential, clinical research is expected to intensify in the coming years, along with dynamic growth in its use in many industrial segments. There are no data in the literature on the use of CBG oil as an active ingredient in polysaccharide films. Therefore, the packaging materials developed in this study, which show great application potential, are a novelty.

Therefore, the aim of this study was to develop and characterize biodegradable polysaccharide-based films composed of pectin and curdlan, enriched with CBG oil, and to evaluate their potential as active packaging materials with antioxidant and antibacterial properties for extending the shelf life and quality of perishable food products such as blackberries.

## 2. Materials and Methods

### 2.1. Preparation of Polysaccharide Films

CBG oil (analyzed by GC-MS) was incorporated into a polysaccharide matrix. Citrus pectin (PC) and curdlan gum (CG) were combined in a 2:1 weight ratio and dissolved in water containing 0.5% (*w*/*w*) glycerin. The mixture was stirred magnetically at 600 rpm for 85 min at 100 °C. CBG oil was then added at different weight percentages: 0.125F, 0.25F, 0.35F, 0.5F, and 0.75F. Each resulting film-forming solution was cast and dried at 22 °C for 48 h to obtain thin films. The films were labeled according to their CBG content as 0F, 0.125F, 0.25F, 0.35F, 0.5F, and 0.75F.

### 2.2. Research of Prepared of Polysaccharide Films

All bio-based packaging films obtained were conditioned at a relative humidity of 50 ± 5% and a temperature of 22 °C prior to analysis. The films were subsequently characterized using a range of analytical techniques, including Fourier Transform Infrared Spectroscopy (FTIR), determination of total polyphenol content (TPC), antioxidant activity assays based on 2,2-diphenyl-1-picrylhydrazyl (DPPH) and 2,2′-azino-bis(3-ethylbenzothiazoline-6-sulfonic acid) (ABTS), mechanical testing performed on a Zwick testing machine (model BDO-FBO 0.5TH), measurement of water vapor transmission rate (WVTR), and color analysis using an EnviSense NR60CP colorimeter in accordance with the Commission Internationale de l′Éclairage (CIE) color system. Microscopic examinations were carried out using an Evo 40 scanning electron microscope (Zeiss, Oberkochen, Germany) and a Zeiss SteREO Discovery.V8 stereo microscope [14,32,33].

### 2.3. Storage of Packaged Blackberries (Black Satin)

**Figure 1:**
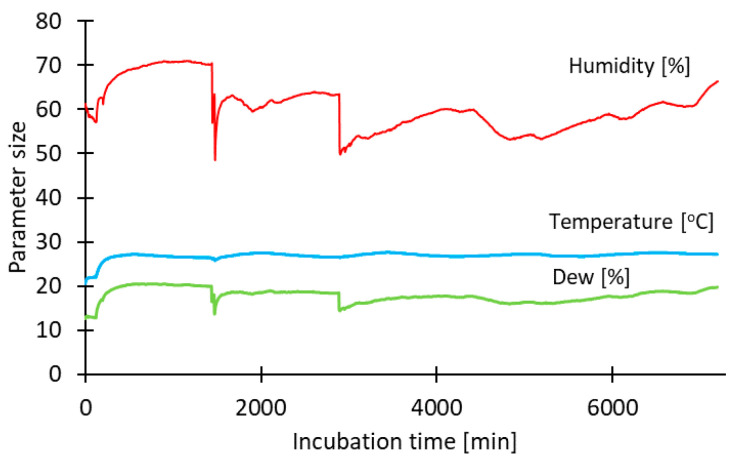
Environmental conditions during the storage of packaged blackberries.

Fresh *Black Satin* blackberries were packed in bags made from the obtained bio-based film, which were sealed using an FKR 200/12 jaw sealer (Kegel Machines, Venray, The Netherlands). The packaged fruits were placed in a chamber covered with black material to prevent exposure to external light. Illumination inside the chamber was provided by a Nature 895 mm/45 W High Lite lamp, ensuring uniform lighting of the packaged samples. The internal conditions of the chamber (temperature, humidity, and dew point) were monitored using a thermohygrometer (Figure 1).

### 2.4. Analysis of Packaged Blackberries (Black Satin)

The anthocyanin content of the packaged fruits was quantified, and the color properties of the fruits were evaluated following the methodology described by Dobrucka et al. [11].

To the weighed (±0.001 g) homogenized blackberries, 10 mL (7.764 g) of methanol was added, thoroughly mixed, and left for 24 h. The extract was then filtered. 0.5 mL of the resulting extract was transferred to a 2 mL Eppendorf tube and 1.5 mL of 0.1% hydrochloric acid in methanol was added. The absorbance of the solution was measured at λ = 528 nm, using a 0.1% solution of hydrochloric acid in methanol as a reference. The anthocyanin content (calculated as mg C3G/100 g fresh fruit, where: “mg C3G” means “mg of cyanidin-3-glucoside equivalents”) was calculated using the following formula:

A—absorbance at 528 nm;

m—mass of the tested plant substance, g

718—specific absorbance of cyanidin 3-O-glucoside chloride at 528 nm

W—conversion factor taking into account sample dilutions

### 2.5. Research on the Application of the Films to the Soil Included the Following Measurements

The vegetation experiment was performed in a climate-controlled chamber at the Faculty of Agriculture, Horticulture, and Bioengineering, Poznan University of Life Sciences. The environmental conditions were maintained at 17 ± 1 °C with 75–80% relative humidity and a 16 h light/8 h dark photoperiod provided by LED lighting at an intensity of 150 µmol·m^−2^·s^−1^. Lettuce seeds (*Lactuca sativa* L. cv. Zeralda; Vilmorin) were sown individually in standard peat substrate. After two weeks, seedlings at the 3–4 leaf stage were transplanted into polyethylene containers with a volume of 1000 cm^3^.

The aim of the study was simply to assess the effect of the films received on the growth of the selected plant and to identify any potential phytotoxicity.

### 2.6. Statistical Analyses

Results are expressed as mean ± standard deviation (SD). Statistical comparisons were carried out via one-way ANOVA using Statistica 13, with significance defined at a 95% confidence interval.

## 3. Results and Discussion

### 3.1. GC-MS of CBG Oil

**Figure 2:**
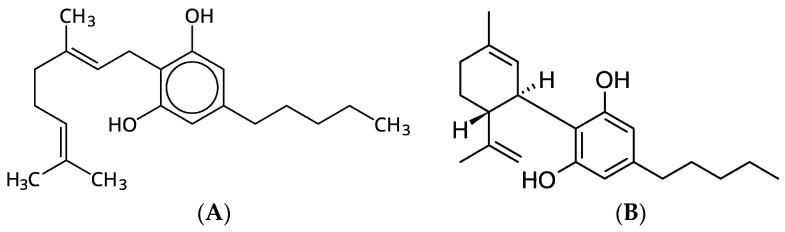
Chemical structure of (**A**) CBG and (**B**) CBD.

Table 1 shows the presence of a number of compounds in CBG oil. Cannabigerol (CBG) (96.70%) and Cannabidiol (CBD) (2.50%) are the compounds (Figure 2) that occur in the highest amounts in the tested oil. For a long time, CBD has been attracting the interest of many scientists due to its high therapeutic potential. CBD, or 2-(6R)-6-isopropenyl-3-methyl-2-cyclohexen-1-yl-5-pentyl-1,3-benzenediol, interacts with specific cannabinoid receptors such as CB1, CB2, and vanilloid receptors, but it is also known to bind non-specifically to more than fifty macromolecular targets in humans, including many enzymes, receptors, ion channels, and transporters [35].

CBD exhibits anti-inflammatory, analgesic, neuroprotective, and anticonvulsant properties, making it a promising therapeutic agent for the treatment of various conditions [36]. CBG, which accounts for as much as 96.70% of the oil used, has many therapeutic, antibacterial, antifungal, and anti-inflammatory properties, and also prevents cell proliferation [37]. Similar to cannabinoids, the complex biological effects of CBG are believed to result from modifications of redox-dependent and inflammatory processes, which in turn modulate cellular metabolism [38]. Due to its broad spectrum of biological activity, CBG appears to be a very promising compound for the treatment of diseases requiring multidirectional pharmacotherapy, which is why we are witnessing its increasingly widespread use due to its beneficial effects on health and lack of psychoactivity.

### 3.2. FTIR of Polysaccharide Films

**Figure 3:**
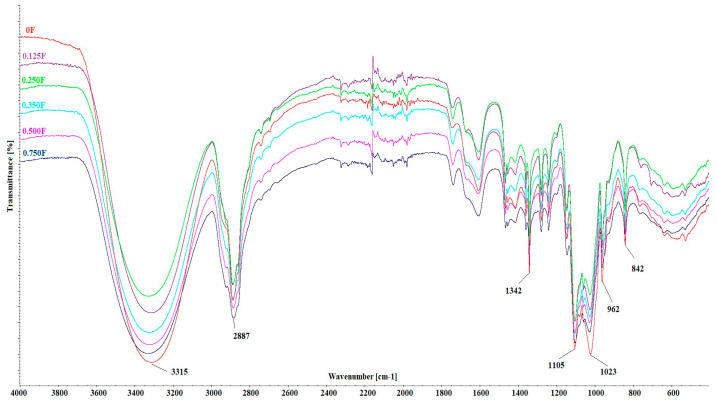
FTIR spectra for polysaccharide films with CBG oil.

Figure 3 shows FTIR spectra for the developed functional polysaccharide films with CBG oil. The following peaks are clearly visible in the spectra: 3315 cm^−1^, 2887 cm^−1^, 1342 cm^−1^, 1105 cm^−1^, 1023 cm^−1^, 962 cm^−1^, 842 cm^−1^. The broad band at 3315 cm^−1^ indicates the presence of hydroxyl groups in both the pectin structure and the CBG molecule, which contains at least two –OH groups [39,40]. Hydroxyl groups are present in both pectin chains and the CBG molecule, indicating the possibility of new hydrogen bonding between these components. The sharp peak at 2887 cm^−1^ is associated with the presence of a long alkyl chain in the cannabigerol (CBG) molecule, confirming the incorporation of oil into the pectin matrix. This is the most pronounced peak for the 0.75F film, which contains the highest amount of CBG oil in the polymer matrix. The signal at 1342 cm^−1^ corresponds to –C–H and –O–H vibrations, while the peaks at 1105 cm^−1^ and 1023 cm^−1^ are typical for glycosidic pectin units. The observed changes in their intensity after the introduction of CBG oil may indicate interfacial interactions that disrupt the ordered network of hydrogen bonds within the polysaccharide. The peaks at 962 cm^−1^ and 842 cm^−1^ are attributed to the vibration of the C–C skeleton, confirming the preservation of the typical pectin structure. The fact that these bands did not undergo significant shifts suggests that there was no degradation of the basic structure of the polysaccharide during oil incorporation [41].

### 3.3. The Study of Polysaccharide Films

**Figure 4:**
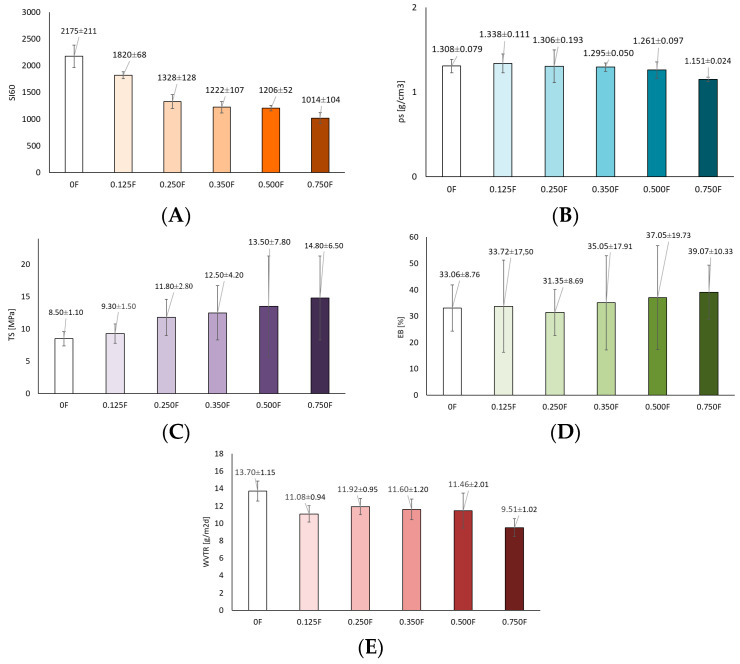
Results of tests on films (**A**) swelling coefficient, (**B**) density, (**C**) tensile strength, (**D**) elongation at break (**E**) WVTR of polysaccharide films.

The study determined the swelling coefficient, density, tensile strength, elongation at break, and WVTR for functional polysaccharide films with CBG oil (Figure 4). It was observed that the swelling coefficient increased with the amount of active ingredient used. The 0F film without additives had an SI60 value of 2175 ± 211. With the amount of CBG oil in the matrices, a linear decrease in the SI60 value was observed: 1820 (0.125F), 1328 (0.25F), 1222 (0.35F), 1206 (0.5F), and 1014 (0.75F). This is the effect of the polysaccharide matrix used: pectin/curdlan gum. Curdlan gum forms ß-helical bonds and strong hydrogen bonds, which causes the film to thicken. Both polysaccharides formed a compact, synergistic polysaccharide network, which consequently causes a decrease in the swelling index and, as a result, greater stability of the film in water. The addition of CBG to the matrix resulted in greater hydrophobicity of the film, which also contributed to a decrease in the swelling index. Therefore, the films obtained, together with the amount of CBG added, became more cross-linked, i.e., they contained less free space for liquid absorption, which is a desirable feature in food films. There were similar results from Tian et al. [42], who studied konjac glucomannan/curdlan films and saw that as the zein content went up, the swelling ratio of the film gradually went down, showing that the hydrophobicity of the additive played a big role. This meant that even if the hydrophobic additive was applied in small amounts, it significantly improved the water resistance of the film. The density of the film is fundamental to understanding the relationship between the molecular structure and the physical properties of the film [43]. For the films obtained, it ranged from 1.308 to 1.151 g/cm^3^. A decrease in density was observed with the amount of CBG added to the matrix. This is the effect of introducing a hydrophobic filler such as CBG oil, which probably not only reduced the hydrophilicity of the film, but most likely caused disturbances in the homogeneity of the structure, including microvoids and small oil bubbles. This relationship, i.e., a lower swelling coefficient and lower density, is consistent with the Flory-Rehner theory of polymer networks, which states that the degree of cross-linking, the size of the network meshes, and the hydrophobic nature of the molecules determine the water absorption capacity and density of the material [44]. With increasing amounts of CBG in the polysaccharide matrix, swelling was reduced and density decreased, indicating less filled and structurally lighter matrices, but with better hydrophobic stability.

In the evaluation of the quality of the manufactured materials, it was necessary to analyze their mechanical and barrier properties to determine whether they are able to withstand external stresses and maintain their integrity as a barrier for the environment and packaged food [45]. The packaging materials must have sufficient mechanical strength and flexibility to withstand the stresses encountered during production and consumer use. Important mechanical properties of packaging films include tensile strength and elongation at break [46,47,48]. TS and EB are two basic properties that are correlated with the chemical structure of the film material [49]. Mechanical properties are related to the structure of the film, where a compact film structure provided relatively better fracture resistance [50]. The issue of additives to the polymer matrix is also important. In this study, it was observed that the addition of CBG oil caused an increase in TS and EB. In these tests, the TS values for the films ranged from 8.50 MPa to 14.80 MPa (for films from 0F to 0.75F). The EB values ranged from 33.06% to 39.07% (for films from 0F to 0.75F). Statistically significant differences (*p* < 0.05) for the tested parameters were observed after the use of higher concentrations of CBG oil, i.e., 0.35F, 0.5F, and 0.75F. This is due to the fact that the resulting pectin/curdlan gum matrix forms a compact network, as confirmed by the SI60 density results. The observed increase in TS caused by the addition of CBG can be attributed to its homogeneous dispersion in the film matrix, which strengthened the network structure of the film through the presence of hydrogen bonds, improving stress transfer throughout the entire volume of the film [51]. The increase in EB is due to the fact that CBG oil can act as a filler/plasticizer, which improves the elasticity of the material without disturbing the integrity of the network. Similar studies were conducted by Meerasri et al. [52], who studied sericin-pectin films with different concentrations of mixed thyme-oregano oil. They obtained TS values ranging from 4.14 ± 0.33 to 5.93 ± 1.01 (at the highest concentration). The barrier properties of food packaging materials are crucial for protecting food from moisture and oxygen, which can cause deterioration and spoilage. The diffusion of gas molecules in films is largely determined by the size of the gas molecules in relation to the available free space in the film and their ability to move through the polymer network [53]. In this study, WVTR values ranged from 13.7 to 9.51 g/m^2^ d for films from 0F to 0.75F. Thus, an increase in CBG oil content improved the barrier properties of the films obtained. This confirms previous studies which confirm that the interaction and compatibility between the polysaccharides used—pectin/ curdlan gum and CBG—lead to denser and more compact films, as well as greater water barrier properties [43].

### 3.4. Study of the Color of Polysaccharide Films

**Figure 5:**
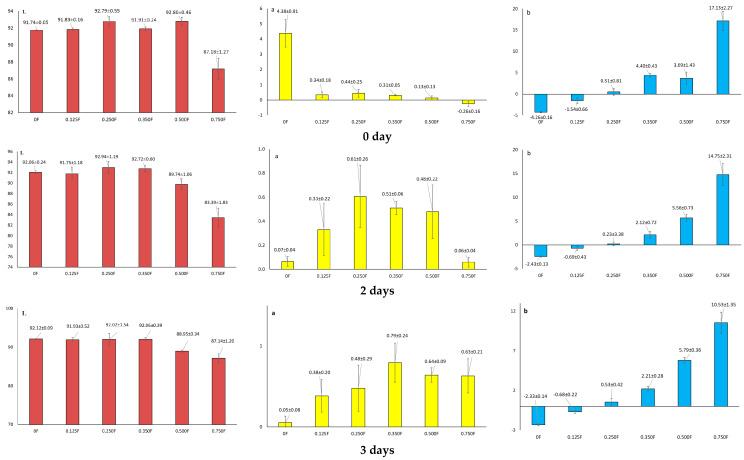

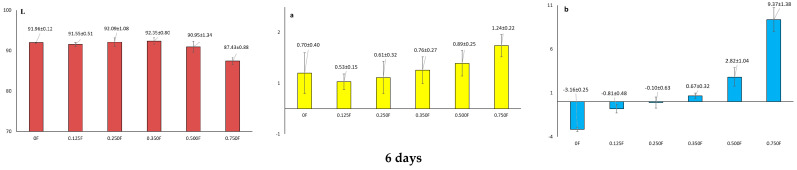
Results L, a, b for the films obtained after 0, 2, 3, and 6 days of storage.

Color plays a critical role in the shelf life of both the packaged product and the packaging material. Moreover, the visual and perceptual attributes of the packaging contribute to the overall evaluation of the product, creating a growing need for packaging designs that effectively stand out among competing items [54]. Because, as we know, packaging is also a silent-seller, the color of the polysaccharide films obtained therefore has a marketing function. Colored films can also play a protective role, as the reduced light permeability of the film can additionally protect packaged food from photo-oxidation [55]. The color of biodegradable/edible films depends on the type, concentration, and size of essential oil droplets, oils, and the transparency of film-forming solutions [56]. In this study, the color parameters of the obtained films were determined: L* (lightness), a* (contribution of red color), and b* (contribution of yellow color). The present study showed that in all tested films, the change in the L* parameter did not change significantly statistically (*p* ≥ 0.05) (Figure 5). In films with low CBG content (0.125F, 0.25F, 0.35F), L* remained stable, indicating their resistance to darkening. The exception was the 0.5F film, in which there was a decrease in L*, suggesting darkening or photodegradation processes. In test 0, the brightness value for the film was 92.80 ± 0.46. After 3 and 6 days, the L value was 88.95 ± 0.34 and 90.95 ± 1.34, respectively. The higher CBG content in the tested films increased the number of potential oxidation sites, resulting in more noticeable darkening of the film over time. L* changes are also related to light diffusion in the polymer structure, modified by CBG. In the case of parameter a*, for 0F film (without CBG), a sudden drop from 4.38 to 0.07 was observed, indicating a loss of red coloration. In the case of films containing CBG, an increase in the proportion of red color was observed during the test. This applied to sample 0.75F, which had the highest content of this component, and the value of a* changed from −0.26 to 1.24, which may indicate the oxidation of CBG in this film. The most dynamic changes in all films concerned the b* parameter. In the case of the 0.75F film, the b* value changed from 17.13 to 9.37, which means a loss of over 45% of the original yellowness, and may result from the decomposition of chromophores in CBG. The high b* value (17.13 ± 2.27) in the 0.75F film in test 0 suggests that CBG oil contributes a strong yellowish coloration, probably related to carotenoids, flavonoids, or cannabigerol isomerization products. Over time, these compounds degraded, reducing the intensity of the yellow color. The observed changes in b* are greatest in films with a high CBG content, confirming its role in color modification.

**Figure 6:**
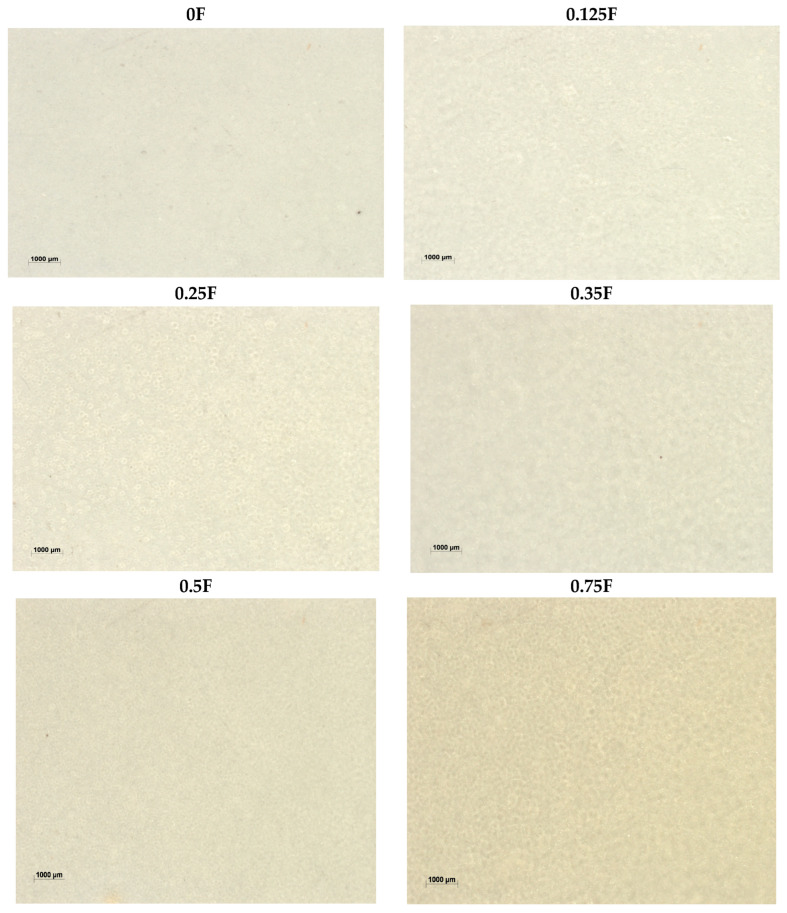
Images of polysaccharide films (0F, 0.125F, 0.25F, 0.35F, 0.5F, 0.75F) made with a stereoscopic microscope.

The observed changes in film color (Figure 6) are proportional to the CBG oil content, confirming its significant impact on film color stability. Changes in the L*, a*, and b* color parameters are correlated with the CBG content in the tested samples. Films containing low concentrations of CBG (0.125F–0.25F) showed the highest color stability. They can therefore be used in products requiring long-term transparency and color stability of the packaging.

### 3.5. Color Testing of Packaged and Unpackaged Fruit

**Figure 7:**
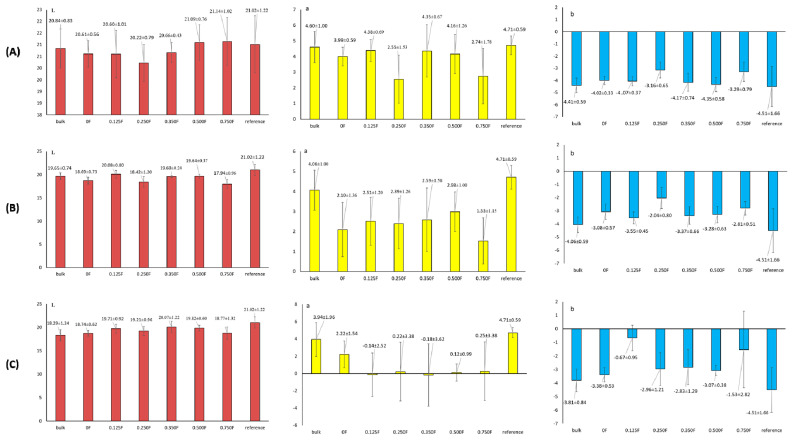
Results L, a, b for stored fruit on days 0, 2 (**A**), 3 (**B**), and 6 (**C**) of the study.

**Figure 8:**
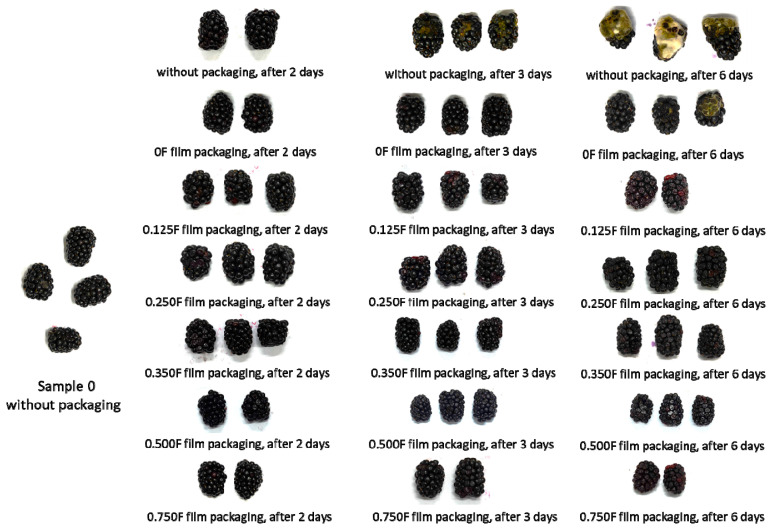
Images of fruit stored without packaging and after packaging in films and stored for 2, 3, and 6 days.

**Figure 9:**
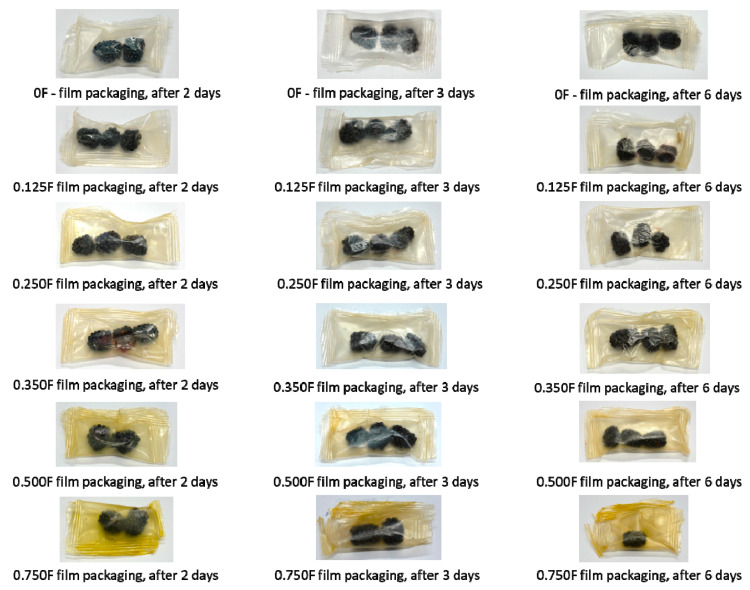
Images of fruit packaged in 0F, 0.125F, 0.25F, 0.35F, 0.5F, and 0.75F film stored for 2, 3, and 6 days.

The color of blueberries and other berries is one of the key attributes affecting perceived quality, freshness, and consumer acceptance [57]. The aim of this study was to determine the effect of films with different concentrations of cannabigerol (CBG) on the color stability of blackberries during storage for 0, 2, 3, and 6 days. Figure 7 shows the L*, a*, and b* values for fruit stored on days 0, 2 (A), 3 (B), and 6 (C) days of the study. The results obtained idicate that storage time significantly affects changes in fruit color, which manifests itself in a decrease in brightness (L*) and shifts in the color scale (a*, b*). The a* parameter, which characterizes the red hue, showed a significant downward trend with storage time. The decrease in a* values indicates a loss of red pigments. The b* values were negative and indicated a predominance of blue-violet hues typical of blackberries. During storage, there was a gradual increase in b* values (i.e., a shift towards less negative values), which indicates a decrease in the intensity of blue-violet colors and lightening of the fruit. This development is also associated with the degradation of anthocyanins and changes in pH in fruit cells. As in the case of a*, the use of film with a higher CBG content had an effect on reducing the loss of b* color intensity, which confirms the stabilizing effect of these bioactive compounds. These changes are further illustrated by pictures (Figure 8) of fruit without packaging and after packaging in films and stored for 2, 3, and 6 days. Mold was observed in fruit without film and packaged in film 0 after 6 days. This phenomenon was not observed in the other films. Films with lower CBG concentrations (0.125F–0.35F) showed the ability to limit these changes, especially in the initial days of storage (up to 3 days), suggesting a protective effect of CBG on fruit color (Figure 9). However, at higher concentrations (0.5F and 0.75F) and longer storage times, increased color changes were observed, which may indicate the pro-oxidative effects of cannabigerol. Higher concentrations of CBG in the films applied to blackberry packaging may have caused pro-oxidative effects, which is consistent with previous reports that some antioxidants may promote the formation of reactive oxygen species under certain conditions and concentrations. In addition, interactions between CBG and other fruit components may lead to pigment destabilization and accelerated color loss. In all variants, especially at higher CBG concentrations in the film, a clear decrease in brightness (L*) is observed. The fruit loses its original intense color, becoming increasingly darker. This effect was probably caused by the significant concentration of anthocyanins in the dry matter of the fruit associated with water removal [58]. It was observed that films containing CBG in concentrations (0.35 F–0.5F) exhibited optimal protective properties.

### 3.6. Research on the Anthocyanin Content of Packaged and Unpackaged Fruit

**Figure 10:**
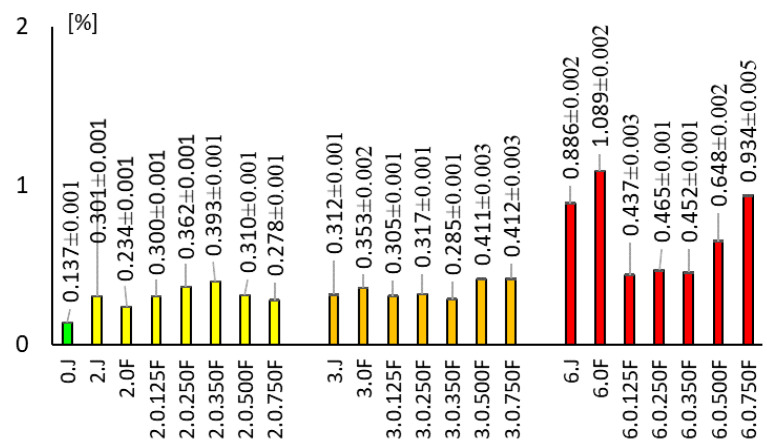
The anthocyanin content of unpacked and packaged fruit after 0, 2, 3, and 6 days of storage.

This study evaluated the effect of the developed films (0F, 0.125F, 0.25F, 0.35F, 0.5F, 0.75F) on the stability of anthocyanins in fruits stored for 2, 3, and 6 days (Figure 10). Fruit stored without film (0.J, 2.J, 3.J) had a lower anthocyanin content compared to most of the fruit packed in film. The use of film without CBG (OF) slowed down the degradation of anthocyanins, which confirms the use of the developed polysaccharide film as an effective barrier against factors causing their degradation (including light). The use of film with added CBG oil produced varying effects depending on the concentration and storage time. In the first days of storage, films with lower CBG concentrations (0.125F and 0.25F) showed similar or slightly higher effectiveness in protecting anthocyanins compared to the classic film. At higher concentrations (≥0.5F), an increase in anthocyanin content was observed over a longer period. The anthocyanin content for 0.5F was 0.648%, while for 0.75F it was 0.934%. The opposite effects were observed in studies on the effect of essential oils and natural extracts in active films. Siracusa et al. [59] showed that adding phenolic plant oils to packaging can improve its antioxidant properties, but at higher concentrations, it can destabilize the matrix and accelerate the degradation of bioactive food components. Similar results were also presented by Kosović et al. [60] also presented similar results when studying the stability of CBD in sunflower oil, where darkening of the samples and a decrease in the active substance content due to oxidation were observed. Films without CBG (0F) show greater color degradation, which confirms the benefits of its use in the polymer matrix. The protective effect of the film against anthocyanins strongly depends on the CBG content in the polysaccharide matrix. The best protective effects and the highest anthocyanin content were observed at CBG concentrations of 0.125F–0.25. They ensured the stabilization of anthocyanins thanks to the antioxidant properties of CBG. Higher CBG concentrations (0.5, 0.75F) in the film matrix after two days of incubation caused a decrease in the anthocyanin content in the fruit, and after 3 and 6 days, an increase compared to other films. The anthocyanin content results correlate with the film color results. Films with higher CBG concentrations (0.5F, 0.75F) also showed a greater increase in anthocyanin content.

### 3.7. SEM Analysis of Polysaccharide Films

**Figure 11:**
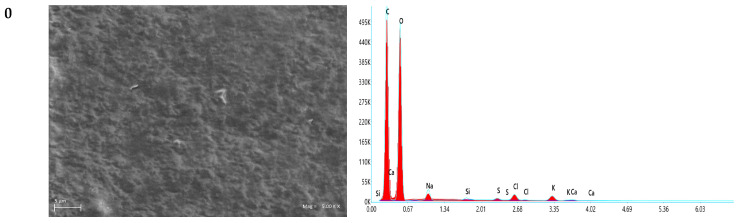

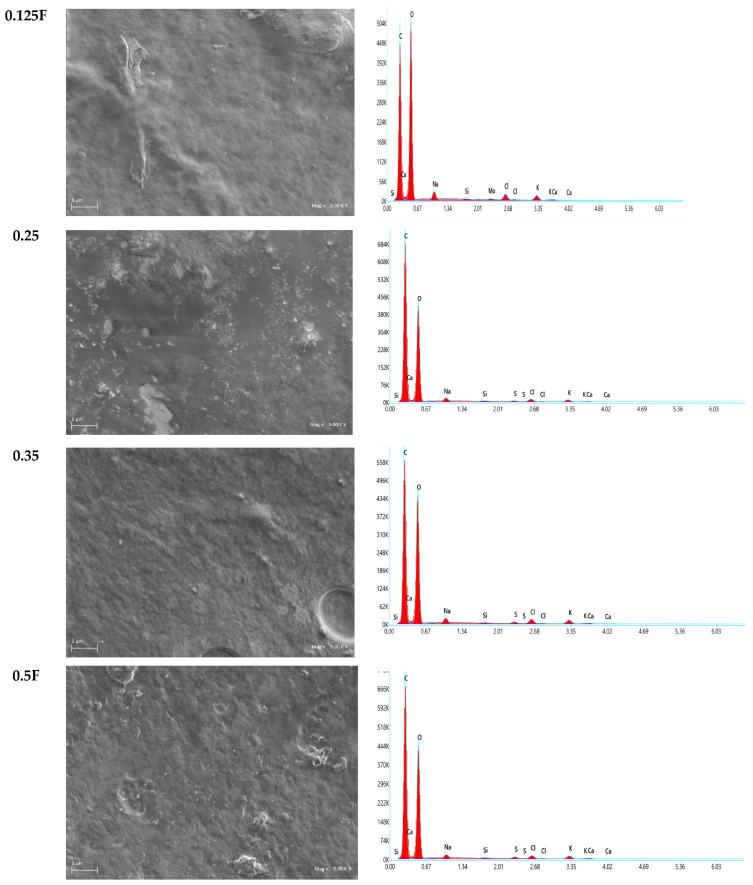

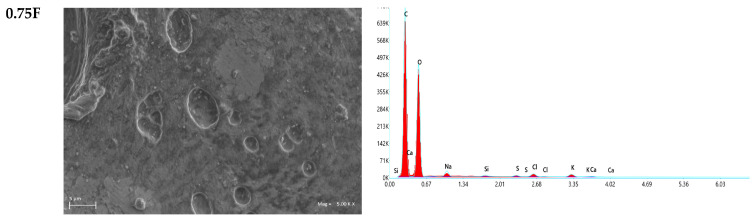
SEM and EDX analysis of functional polysaccharide films with CBG oil.

For evaluation of the film morphology and distribution of CBG oil in the film matrix, a scanning electron microscope (Figure 11) was used, which allowed us to better understand the mechanical properties and water vapor transmission mechanisms of the obtained films. SEM and associated EDX images confirm that the films obtained are free of contaminants or toxic metals, which confirms their suitability for use in food packaging. The surface of the 0F sample appears smooth, without cracks or signs of phase separation, which indicates consistent and strong interactions within the polymer matrix. The addition of CBG oil at lower concentrations (0.125F, 0.25F) did not affect the surface of the film. However, a higher oil content in the tested films resulted in visible changes on the film surface, such as indentations and roughness. The roughness of the film surface can be attributed to emulsification, flocculation, and coalescence during the drying process, which leads to the formation of droplets and the volatilization of CBG [61]. Ji et al. [38] obtained composite chitosan films based on tea tree oil nano-microcapsules and observed agglomeration and coalescence of TSO, as well as a rough surface of the CS/PC/TSO layers, which affected the properties of the obtained films. The observations obtained in this study (visible defects) correspond to the physicochemical results, i.e., the observed decrease in density (empty spaces) and decrease in swelling (less surface area for water).

### 3.8. Results of Vegetation Experiment

A clear differentiation was observed among the experimental variants in terms of lettuce head dry matter content, which serves as an indicator of yield. The lowest dry matter value was recorded in the 0.25F, 1 g treatment, whereas the highest was observed in the 0.75F, 2 g variant. In all combinations except one (0.25F, 1 g), the addition of the foil to the substrate resulted in an increase in lettuce yield compared with the control plants. In the variant where foil without the active substance (0F, 1 g) was applied, a higher dry matter content was also noted compared with the control. The obtained results indicate a complex influence of both the concentration of the extract incorporated into the foil and the amount of foil added to the substrate on the productivity of lettuce plants.

The leaf water content of lettuce, expressed as Relative Water Content (RWC), varied depending on the applied foil treatment. The lowest RWC value was recorded in the 0.75F, 1 g combination, reaching 85.61%. In contrast, the highest RWC value was observed in the 0.5F, 2 g variant, amounting to 96.95%, which indicates a high level of leaf hydration. In all treatments except one (0.75F, 1 g), higher RWC values were observed in lettuce grown in substrates supplemented with foil compared with the control plants. Across all tested concentrations except 0.35F, higher RWC values were recorded for the 2 g application rate compared with the 1 g variants.

The total chlorophyll content in lettuce leaves varied depending on the concentration of the active substance in the substrate. In the control samples, the values ranged from 0.301 to 0.456 mg·g^−1^ fresh weight (FW). At a low concentration of the active substance (0.125F), the total chlorophyll level was comparable to or slightly higher than the control (0.330–0.552 mg·g^−1^ FW). In the case of foil applications at concentrations of 0.25 and 0.350, a marked increase in chlorophyll a and b content was observed, particularly in the variants where 2 g of foil was applied (up to 0.682 mg·g^−1^ FW). As a result of substrate treatment with the highest concentration of the active substance (0.75F), the chlorophyll content decreased to 0.260–0.590 mg·g^−1^ FW. When comparing treatments where 1 g and 2 g of foil were applied within the same concentration of active substances released from the foil, noticeable differences were found mainly in the 0.25 and 0.35 variants, where an increase in the applied foil mass resulted in higher total chlorophyll concentration.

The carotenoid content in lettuce leaves ranged from 0.054 to 0.132 mg·g^−1^ fresh weight (FW), while in the control variants it was between 0.059 and 0.093 mg·g^−1^ FW. A low concentration of active compounds in the foil (0.125F) did not significantly affect carotenoid levels. At moderate concentrations (0.25, 0.35F, and 0.5F), an increase in carotenoid pigment content was observed, with the most pronounced rise recorded in variants where 2 g of foil were applied. At the highest concentration of active compounds in the foil (0.75F), the carotenoid content decreased to 0.054–0.113 mg·g^−1^ FW, and the differences between 1 g and 2 g applications were less distinct.

The level of superoxide anion radical in the control samples ranged from 1.103 to 1.463 A·g^−1^ fresh weight (FW). Low and moderate concentrations of active substances in the foil (0.125F, 0.25, 0.35F) led to an increase in this parameter. This trend was particularly evident in the variants where 1 g of foil was applied. In the treatments where 2 g of foil were used at the same concentration, the O_2_•^−^ values were generally lower than those observed for the 1 g applications. The highest superoxide anion level was recorded in the 0.25 variant.

The phenolic content in lettuce leaves ranged from 1546.96 to 5214.10 µg·g^−1^ fresh weight (FW). In the control samples, the values were between 1793 and 3990 µg·g^−1^ FW. Low concentrations of the active substance in the foil (0.125F) caused a slight increase in phenolic compound content, whereas at moderate concentrations (0.25–0.35F), the accumulation of these compounds in lettuce leaf blades was markedly higher. A greater amount of foil (2 g) within the same concentration level led to enhanced phenolic accumulation. At the highest concentration of the active substance in the foil (0.75F) applied at 2 g, a substantial increase in phenolic compound levels was recorded.

The flavonol content in control lettuce plants ranged from 0.45 to 0.59 µg·g^−1^ fresh weight (FW). Low concentrations of the active substance in the foil did not significantly affect the levels of these compounds. The application of moderate concentrations of active substances (0.25–0.35F) resulted in an increase in flavonol content, and a higher amount of foil (2 g) within the same concentration led to elevated flavonol levels in leaf blades, reaching up to 0.71 µg·g^−1^ FW. At the highest concentration of active substances in the foil (0.75F), the flavonol content was lower, and the differences between the 1 g and 2 g treatments were not substantial.increased.

The application of low concentrations of active substances resulted in only minor changes in the level of free amino acids, whereas the use of moderate and high concentrations of the active compounds incorporated into the foils led to an increase in the accumulation of free amino acids in lettuce leaf tissues, particularly in the 2 g treatments within the same concentration range. The lowest content of free amino acids was recorded in the 0.125F variant, while the highest levels were observed in the 0F (1 g and 2 g) and 0.35F (2 g) variants.

In the control variants, the carbohydrate content ranged from 335 to 612 µg·g^−1^ FW. Low concentrations of the foil caused a moderate increase in carbohydrate accumulation. In the 0.25F, 0.35F, and 0.5F variants, a pronounced increase in carbohydrate levels was observed, particularly in treatments where 2 g of foil was applied. At the highest concentration (0.75F), carbohydrate content was lower in the 2 g treatments but higher in the 1 g treatments, indicating a complex, dose-dependent response of the plants.

**Figure 12:**
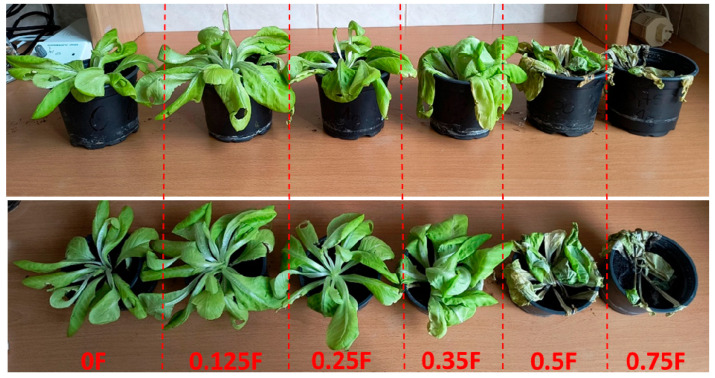
The salad plants planted with 1 g of foil at concentrations ranging from 0F to 0.75F, from left to right.

Figure 12 shows salad plants planted with 1 g of foil at concentrations ranging from 0F to 0.75F, from left to right. Observed changes in relative water content (RWC), dry matter, and photosynthetic pigments in lettuce heads indicate a complex physiological and biochemical response of plants to soil-applied films containing bioactive substances. Variants with low and moderate concentrations of active compounds promoted higher RWC, chlorophyll, and carotenoid levels, suggesting a positive effect on water status and photosynthetic processes. This phenomenon may result from the stimulation of enzymes involved in chlorophyll biosynthesis and enhanced electron transport efficiency in the light phase of photosynthesis. Similar trends were observed in *Lactuca sativa* treated with humic substances as biostimulants mitigating drought stress, where plants treated with humic substances showed improved net photosynthetic rate, carboxylation efficiency, electron transport flux, and water use efficiency [62]. Likewise, application of microalgae as a source of growth-promoting bioactive compounds increased chlorophyll content by 10% compared to controls [63].

The lowest RWC and reduced chlorophyll content in the variant with the highest concentration (0.75F, 1 g) may indicate oxidative stress and reduced photosynthetic activity. Excess active substances could disrupt chloroplast membrane integrity or limit the expression of genes involved in pigment biosynthesis. This phenomenon resembles the typical plant response to excessive doses of growth regulators or soil-applied biostimulants, where beneficial effects observed at low doses are reversed due to the toxic action of excessive active compounds. Similar phytotoxic effects caused by high biostimulant doses in lettuce were reported by Velasco-Clares et al. [64].

**Figure 13:**
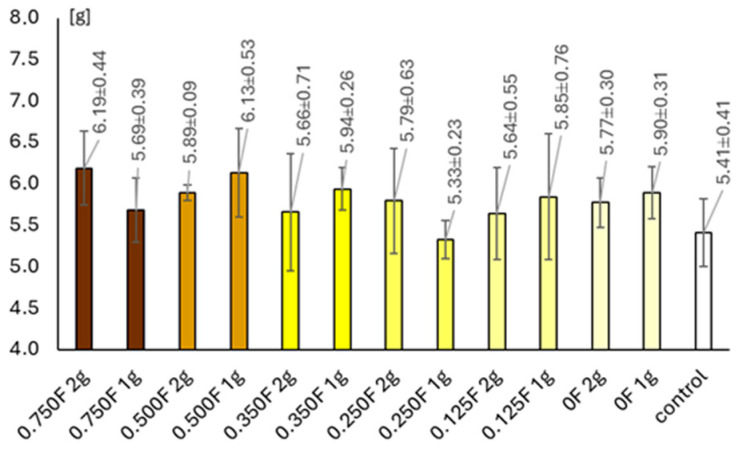
The relative water content (RWC).

The variant with moderate active substance concentration (0.5F, 2 g) exhibited the highest RWC (Figure 13) and elevated chlorophyll and carotenoid content (Table 2), suggesting effective regulation of water balance and photosynthetic activity. Higher carotenoid levels may reflect an antioxidant adaptation, as these compounds scavenge reactive oxygen species (ROS) and protect membrane structures from oxidative damage [65,66]. Temperature reports indicate that mild metabolic stress can trigger increased carotenoid synthesis, stabilizing photosynthetic processes [67].

**Figure 14:**
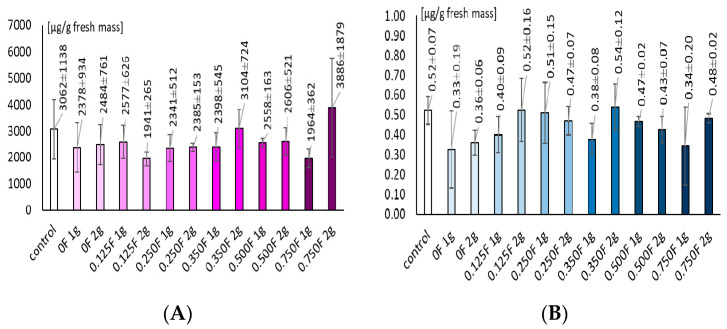
Contents of phenols (**A**) and flavonols (**B**).

High phenolic and flavonol content (Figure 14) in lettuce leaves at moderate film concentrations confirms the activation of phenylpropanoid pathways. These compounds act as potent antioxidants, protecting cell membranes from lipid peroxidation and neutralizing free radicals [68]. Their increase may be interpreted as a mild stress effect acting as a priming factor, preparing plants for potential stress conditions. Similar effects were reported in lettuce treated with soil-applied extracts, where enhanced phenolic and flavonoid synthesis was associated with activation of defense mechanisms and improved photosynthetic performance [69].

**Figure 15:**
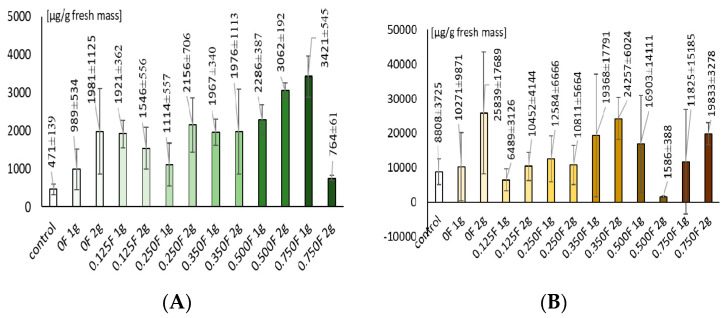
Carbohydrate content (**A**) and free amino acid content (**B**).

Changes in primary metabolism, including increased free amino acids and soluble carbohydrates (Figure 15), indicate activation of osmoregulatory mechanisms. Under mild metabolic stress, plants accumulate osmoprotectants such as proline, glycine, and soluble sugars, which stabilize protein and membrane structures and help maintain cellular water potential [70]. In the present study, the increase of these metabolites coincided with higher phenolic and carotenoid content, suggesting a coordinated metabolic response typical for the action of mild soil-applied biostimulants.

**Figure 16:**
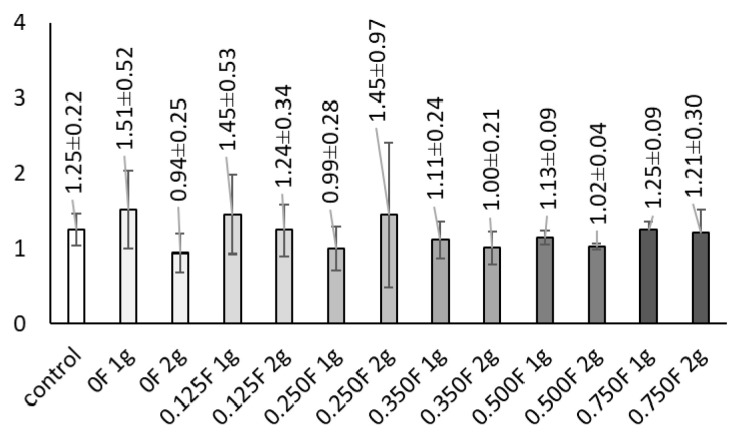
Content superoxide anion radical.

The highest dry matter of lettuce heads, observed in the 0.75F 2 g variant, may result from enhanced photosynthetic activity and increased carbohydrate synthesis under moderate stress conditions. This hormetic effect—stimulation of growth and metabolism at low stressor doses and inhibition at high doses—has been repeatedly reported in the context of biostimulants [71,72]. Figure 16 presents the content superoxide anion radical.

Overall, the soil-applied bioactive films induced a complex physiological and metabolic response in lettuce, including activation of antioxidant, osmoregulatory, and photosynthetic pathways. Applied doses differentially influenced the intensity of responses, with low and moderate doses stimulating secondary metabolism and photosynthetic processes, whereas the highest concentrations had inhibitory effects. These results confirm that the effect of biostimulants or bioactive compound-containing materials is strongly dose- and composition-dependent, which has important implications for cultivation practices and the development of novel plant bioproducts.

## 4. Conclusions

In this study, polysaccharide films based on a pectin/gum curdlan matrix with the addition of (CBG) were developed. Detailed physicochemical, mechanical, and barrier characteristics were determined, and the effect of the films on the color and anthocyanin stability in stored *Black Satin* blackberries was evaluated. A comprehensive assessment of the morphology, physicochemical properties, and effect of bioactive films containing CBG oil on plant physiology was also performed. SEM/EDX analysis showed that the films had a homogeneous structure. It was noted that an increase in CBG oil content caused a significant increase in the hydrophobicity of the films, which manifested itself in a decrease in the swelling coefficient and density, as well as an improvement in mechanical properties and moisture vapor transmission rate (WVTR). Films with lower CBG concentrations showed more stable color and better protection of anthocyanins in fruit during short-term storage, while higher CBG concentrations led to intensification of processes causing color changes and an increase in anthocyanin content. Optimal protective properties and color and anthocyanin stability in fruit were achieved at moderate CBG concentrations (0.125F–0.35%), indicating their potential for use in the short-term storage of products with high sensory value. The higher concentrations of CBG oil can cause a pro-oxidative effect, limiting the durability of natural dyes and reducing the visual appeal of packaging. The study of the biological impact of the film on lettuce plants showed that the effect depended mainly on the concentration of the active substance. Moderate doses (0.25F–0.35F) stimulated the accumulation of chlorophylls, carotenoids, phenols, flavonols, free amino acids, and carbohydrates, indicating the activation of photosynthetic processes and defense mechanisms. In contrast, the highest concentration (0.75F) resulted in a decrease in pigment and metabolite levels, suggesting mild oxidative stress. Changes in O_2_•^−^ levels confirm a short-term stress response, the intensity and course of which depended on the dose and weight of the applied film. The results obtained indicate a complex relationship between the structure and composition of the films and their biological activity. A properly optimized concentration of CBG oil allows for the production of films with beneficial mechanical properties and potential biostimulating effects, which opens up prospects for their use not only in food packaging, but also in agriculture and plant protection.

## Figures and Tables

**Table 1:** Chemical compounds of CBG oil.

Peak RT (min)	Area	Quantity (%)	Compound Name	Formula	*m*/*z*
21,518	2.86 × 10^6^	0.18	Pregna-20-ol-3-on-19-oic acid lactone	C_21_H_30_O_3_	72.9; 82.9; 242.8; 258.5; 259.3
21,995	1.86 × 10^6^	0.12	β-Ionone	C_13_H_20_O	55.1; 66.9; 79.0; 80.7; 206.9
22,044	1.58 × 10^6^	0.10	Trilinolein	C_57_H_98_O_6_	54.8; 67.2; 79.2; 82.9; 206.8
22,107	4.29 × 10^6^	0.27	Olean-12-ene-3ß,15a,16a,21ß,22a,28-hexol	C_30_H_50_O_6_	55.0; 69.2; 83.9; 207.0; 280.0
23,469	2.31 × 10^6^	0.14	7,10,13-Eicosatrienoic acid, methyl ester	C_21_H_36_O_2_	55.1; 67.0; 78.9; 81.1; 207.0
23,735	4.03 × 10^7^	2.50	Cannabidiol	C_21_H_30_O_2_	76.9; 174.2; 206.9; 231.0; 232.2
25,213	1.56 × 10^9^	96.70	Cannabigerol	C_21_H_32_O_2_	68.9; 122.9; 193.0; 194.1; 231.0

**Table 2:** Chlorophyll and carotenoid content in salad leaves after application of the tested films to the soil.

Sample	Chl a (mg/g FW)	Chl b (mg/g FW)	Chl tot (mg/g FW)	Car (mg/g FW)
control	0.287 ± 0.065	0.115 ± 0.024	0.401 ± 0.087	0.079 ± 0.018
0F 1 g	0.267 ± 0.022	0.106 ± 0.009	0.373 ± 0.026	0.075 ± 0.004
0F 2 g	0.343 ± 0.023	0.140 ± 0.015	0.482 ± 0.036	0.093 ± 0.011
0.125F 1 g	0.324 ± 0.083	0.134 ± 0.032	0.459 ± 0.115	0.090 ± 0.019
0.125F 2 g	0.342 ± 0.046	0.142 ± 0.019	0.485 ± 0.065	0.091 ± 0.009
0.25F 1 g	0.350 ± 0.057	0.135 ± 0.033	0.485 ± 0.089	0.092 ± 0.018
0.25F 2 g	0.347 ± 0.093	0.139 ± 0.040	0.486 ± 0.134	0.093 ± 0.023
0.35F 1 g	0.401 ± 0.036	0.154 ± 0.014	0.555 ± 0.050	0.102 ± 0.011
0.35F 2 g	0.312 ± 0.081	0.120 ± 0.025	0.432 ± 0.105	0.087 ± 0.021
0.5F 1 g	0.386 ± 0.128	0.153 ± 0.062	0.539 ± 0.190	0.104 ± 0.038
0.5F 2 g	0.287 ± 0.034	0.115 ± 0.015	0.402 ± 0.048	0.082 ± 0.004
0.75F 1 g	0.335 ± 0.104	0.118 ± 0.035	0.453 ± 0.139	0.081 ± 0.019
0.75F 2 g	0.318 ± 0.119	0.126 ± 0.050	0.443 ± 0.168	0.087 ± 0.030

## Data Availability

The original contributions presented in this study are included in the article. Further inquiries can be directed to the corresponding authors.

## References

[1] Gryszczyńska B., Iskra M., Gryszczyńska A., Budzyń M. (2011). Aktywność przeciwutleniająca wybranych owoców jagodowych. Postępy Fitoter..

[2] Koca I., Karadeniz B. (2009). Antioxidant properties of blackberry and blueberry fruits grown in the Black Sea Region of Turkey. Sci. Hortic..

[3] González-Barrio R., Truchado P., Ito H., Espín J.C., Tomás-Barberán F.A. (2011). UV and MS identification of urolithins and nasutins, the bioavailable metabolites of ellagitannins and ellagic acid in different mammals. J. Agric. Food Chem..

[4] Pantelidis G.E., Vasilakakis M., Manganaris G.A., Diamantidis G.R. (2007). Antioxidant capacity, phenol, anthocyanin and ascorbic acid contents in raspberries, blackberries, red currants, gooseberries and Cornelian cherries. Food Chem..

[5] Song H., Asghari M., Zahedipour-Sheshglani P., Alizadeh M., Qian S., Diao E. (2023). Modeling and optimizing the effects of Trichoderma on quality, decay extension rate and phytochemical compounds of Thompson seedless table grapes by the use of response surface methodology. Eur. J. Agron..

[6] Dobrucka R., Szymański M. (2025). Bio-based pectin films with cannabidiol extract from pollen of *Cannabis sativa* L.—Active packaging to protect anthocyanins in *Thomson seedless* dark grapes. Colloids Surf. B Biointerfaces.

[7] Jakrawatana N., Ngammuangtueng P., Vorayos N., Gheewala S.H. (2023). Replacing single-use plastics with bio-material packaging in Thailand and impacts on the water–energy–climate Nexus. Sustain. Prod. Consum..

[8] Teixeira N. (2025). Circular economy perspectives: Challenges, innovations, and sustainable futures. Discov. Sustain..

[9] Mehran M., Huang L., Geng M., Gan Y., Cheng J., Zhu Q., Mustafa A. (2025). Co-utilization of green manure with straw return enhances the stability of soil organic carbon by regulating iron-mediated stabilization of aggregate-associated organic carbon in paddy soil. Soil Tillage Res..

[10] Dobrucka R., Urbaniak M., Kozak W., Szymański M. (2024). Innovative method of environmental safety research of starch-based films with silver nanoparticles. Environ. Prog. Sustain. Energy.

[11] Dobrucka R., Vapenka L., Pawlik M., Szymański M. (2026). In situ Ca^2+^-cross-linking effects on pectin–tragacanth films for fresh raspberry preservation. Colloids Surf. B Biointerfaces.

[12] Upadhyay V., Choudhary K.K., Agrawal S.B. (2024). Use of biochar as a sustainable agronomic tool, its limitations and impact on environment: A review. Discov. Agric..

[13] Dobrucka R., Pawlik M., Szymański M. (2024). Green packaging films with antioxidant activity based on pectin and *Camellia sinensis* leaf extract. Molecules.

[14] Dobrucka R., Vapenka L., Szymański M., Pawlik M., Lasik-Kurdyś M., Gumienna M. (2025). Bio-packaging based on pectin/tragacanth gum with added extracts of cherry waste from the wine industry as a new generation of active films for the food industry. Foods.

[15] Szymański M., Długaszewska J., Pawlik M., Dobrucka R. (2024). Development of innovative environmental safety: Bioactives against pathogenic bacteria red pectin films from *Hibiscus sabdariffa* flos extract for circular economy. Films.

[16] Yanat M., Schroën K. (2025). Bio-nanocomposites as future food packaging materials: A multi-faceted comparison. React. Funct. Polym..

[17] Ren S., Wang T., Guenet B., Liu D., Cao Y., Ding J., Piao S. (2024). Projected soil carbon loss with warming in constrained Earth system models. Nat. Commun..

[18] Babaei Rad S., Mumivand H., Mollaei S., Khadivi A. (2025). Effect of drying methods on phenolic compounds and antioxidant activity of *Capparis spinosa* L. fruits. BMC Plant Biol..

[19] Zhao X., Ma X., Chen B., Shang Y., Song M. (2022). Challenges toward carbon neutrality in China: Strategies and countermeasures. Resour. Conserv. Recycl..

[20] Bhaswant M., Shanmugam D.K., Miyazawa T., Abe C., Miyazawa T. (2023). Microgreens—A comprehensive review of bioactive molecules and health benefits. Molecules.

[21] Mir S.A., Shah M.A., Mir M.M. (2017). Microgreens: Production, shelf life, and bioactive components. Crit. Rev. Food Sci. Nutr..

[22] Shashidhara V., Alwarsamy M. (2024). Pectin nanoforms—A multifaceted polysaccharide and a propitious nanocarrier for medical ailments. Polym. Bull..

[23] Suhartoyo A., Chelminiak-Dudkiewicz D., Ziegler-Borowska M. (2025). Unlocking natural cross-link agents for biopolymer wound dressings: A review. Ind. Crops Prod..

[24] Mellinas C., Ramos M., Jiménez A., Garrigós M.C. (2020). Recent trends in the use of pectin from agro-waste residues as a natural-based biopolymer for food packaging applications. Materials.

[25] Hosseini Abari A., Amini Rourani H., Ghasemi S.M., Kim H., Kim Y.G. (2021). Investigation of antioxidant and anticancer activities of unsaturated oligo-galacturonic acids produced by pectinase of Streptomyces hydrogenans YAM1. Sci. Rep..

[26] Aquinas N., Bhat M.R., Selvaraj S. (2022). A review presenting production, characterization, and applications of biopolymer curdlan in food and pharmaceutical sectors. Polym. Bull..

[27] Yuan M., Fu G., Sun Y., Zhang D. (2021). Biosynthesis and applications of curdlan. Carbohydr. Polym..

[28] Zhang L., Yue L.N., Qian J.Y., Ding X.L. (2020). Effect of curdlan on the rheological properties of hydroxypropyl methylcellulose. Foods.

[29] Wu J., Jiao Y., Yu W., Zhang Y., Li Z., Wang X. (2025). Preparation of chitosan quaternary ammonium salt/pectin antifogging and antibacterial composite film loaded with riboflavin and its application in rape preservation. Food Chem..

[30] Bhatia S., Al-Harrasi A., Shah Y.A., Alrasbi A.N.S., Jawad M., Koca E., Mohan S. (2024). Structural, mechanical, barrier and antioxidant properties of pectin and xanthan gum edible films loaded with grapefruit essential oil. Heliyon.

[31] Biratu G., Gonfa G., Bekele M., Woldemariam H.W. (2024). Extraction and characterization of pectin from coffee (*Coffea arabica* L.) pulp obtained from four different coffee producing regions. Int. J. Biol. Macromol..

[32] Szymański M., Studzińska-Sroka E., Paczkowska-Walendowska M., Gumienna M., Lasik-Kurdyś M., Dobrucka R. (2025). Novel eco-friendly active polysaccharides packaging materials according *E. coli* based on herbal raw material (*Rumex hydrolapathum*) extract for environmental and consumer safety. Cellulose.

[33] Dobrucka R., Dlugaszewska J., Pawlik M., Szymański M. (2025). Innovative active bio-based food packaging material with *Cannabis sativa* L. seeds extract as an agent to reduce food waste. Colloids Surf. B Biointerfaces.

[34] Borrelli F., Fasolino I., Romano B., Capasso R., Maiello F., Coppola D., Izzo A.A. (2013). Beneficial effect of the non-psychotropic plant cannabinoid cannabigerol on experimental inflammatory bowel disease. Biochem. Pharmacol..

[35] Štern A., Novak M., Kološa K., Trontelj J., Žabkar S., Šentjurc T., Žegura B. (2024). Exploring the safety of cannabidiol (CBD): A comprehensive in vitro evaluation of the genotoxic and mutagenic potential of a CBD isolate and extract from *Cannabis sativa* L.. Biomed. Pharmacother..

[36] Yang F., Duan S., Liu J., An Z., Liu W., Wang X., Yang L. (2025). Antitumor effects of cannabidiol (CBD) on osteosarcoma by targeting TNF-α/NF-κB/CCL5 signaling axis. Phytomedicine.

[37] Cabrera C.L.R., Keir-Rudman S., Horniman N., Clarkson N., Page C. (2021). The anti-inflammatory effects of cannabidiol and cannabigerol alone, and in combination. Pulm. Pharmacol. Ther..

[38] Ji Q., Jin Z., Ding W., Wu Y., Liu C., Yu K., Zhang N., Jin G., Lu P., Bao D. (2023). Chitosan composite films based on tea seed oil nano-microcapsules: Antibacterial, antioxidant and physicochemical properties. Food Packag. Shelf Life.

[39] Pirsa S., Aghbolagh Sharifi K. (2020). A review of the applications of bioproteins in the preparation of biodegradable films and polymers. J. Chem. Lett..

[40] Rani S., Lal S., Kumar S., Kumar P., Nagar J.K., Kennedy J.F. (2024). Utilization of marine and agro-waste materials as an economical and active food packaging: Antimicrobial, mechanical and biodegradation studies of o-carboxymethyl chitosan/pectin/neem composite films. Int. J. Biol. Macromol..

[41] da Silva Sasaki J.C., Su Y., Spinosa W.A., de Lima Lopes Filho P.E., Burd B.S., Scontri M., Herculano R.D. (2025). Eco-sustainable, edible, biodegradable and antioxidant pectin and bacterial cellulose films loaded with coconut oil for strawberry preservation. Int. J. Biol. Macromol..

[42] Tian R., Yuan S., Jiang J., Kuang Y., Wu K., Sun S., Jiang F. (2024). Improvement of mechanical, barrier properties, and water resistance of konjac glucomannan/curdlan film by zein addition and the coating for cherry tomato preservation. Int. J. Biol. Macromol..

[43] Karki S., Kim H., Na S.J., Shin D., Jo K., Lee J. (2016). Thin films as an emerging platform for drug delivery. Asian J. Pharm. Sci..

[44] Singh A., Sharma P.K., Garg V.K., Garg G. (2010). Hydrogels: A review. Int. J. Pharm. Sci. Rev. Res..

[45] Singh B., Sharma V., Chauhan D. (2010). Gastroretentive floating sterculia–alginate beads for use in antiulcer drug delivery. Chem. Eng. Res. Des..

[46] Ranasinghe H. (2022). Carbon net-zero by 2050: Benefits, challenges and way forward. J. Trop. For. Environ..

[47] Afzia N., Bora S., Ghosh T. (2025). Utilization of cassava peel based cellulose nanofiber for developing functionalized pectin/pullulan/olive oil nanocomposite film for cling wrapping of chicken meat. Int. J. Biol. Macromol..

[48] Versino F., Ortega F., Monroy Y., Rivero S., López O.V., García M.A. (2023). Sustainable and bio-based food packaging: A review on past and current design innovations. Foods.

[49] Jastrząb A., Jarocka-Karpowicz I., Skrzydlewska E. (2022). The origin and biomedical relevance of cannabigerol. Int. J. Mol. Sci..

[50] Ghaderi P., Najafi M.A., Soltani Tehrani N. (2023). The effect of antibacterial edible film based on sodium caseinate-nanocrystal cellulose containing cells and supernatant of Lactobacillus reuteri on quality of kebab. J. Food Sci. Technol..

[51] Meerasri J., Sukatta U., Rugthaworn P., Klinsukhon K., Khacharat L., Sakayaroj S., Sothornvit R. (2024). Synergistic effects of thyme and oregano essential oil combinations for enhanced functional properties of sericin/pectin film. Int. J. Biol. Macromol..

[52] Liu Z., Lin D., Shen R., Yang X. (2020). Characterizations of novel konjac glucomannan emulsion films incorporated with high internal phase Pickering emulsions. Food Hydrocoll..

[53] Xue W., Zhu J., Sun P., Yang F., Wu H., Li W. (2023). Permeability of biodegradable film comprising biopolymers derived from marine origin for food packaging application: A review. Trends Food Sci. Technol..

[54] Mohammadian M., Salami M., Moghadam M., Amirsalehi A., Emam-Djomeh Z. (2021). Mung bean protein as a promising biopolymeric vehicle for loading of curcumin: Structural characterization, antioxidant properties, and in vitro release kinetics. J. Drug Deliv. Sci. Technol..

[55] Zhou Y., Wu W., Hileuskaya K., Shao P. (2025). Oriented structure design of pectin/Ag nanosheets film with improved barrier and long-term antimicrobial properties for edible fungi preservation. Food Chem..

[56] Hasheminya S.M., Dehghannya J. (2021). Development and characterization of novel edible films based on *Cordia dichotoma* gum incorporated with *Salvia mirzayanii* essential oil nanoemulsion. Carbohydr. Polym..

[57] Pasławska M., Stępień B., Jałoszyński K. (2010). Zmiany parametrów barwy owoców jagodowych wywołane suszeniem, przechowywaniem i rehydracją. Inżynieria Rol..

[58] Pasławska A., Pełka A. (2006). Właściwości rekonstytucyjne i barwa suszu truskawkowego. Żywność Nauka Technol. Jakość.

[59] Siracusa R., Scuto M., Fusco R., Trovato A., Ontario M.L., Crea R., Di Paola R., Cuzzocrea S., Calabrese V. (2020). Anti-inflammatory and anti-oxidant activity of Hidrox^®^ in rotenone-induced Parkinson’s disease in mice. Antioxidants.

[60] Kosović E., Sýkora D., Kuchař M. (2021). Stability Study of Cannabidiol in the Form of Solid Powder and Sunflower Oil Solution. Pharmaceutics.

[61] Bhatia S., Al-Harrasi A., Al-Azri M.S., Ullah S., Bekhit A.E.D.A., Pratap-Singh A., Aldawsari M.F. (2022). Preparation and physiochemical characterization of bitter orange oil loaded sodium alginate and casein based edible films. Polymers.

[62] Atero-Calvo S., Magro F., Masetti G., Navarro-León E., Blasco B., Ruiz J.M. (2025). Potential for drought stress alleviation in lettuce (*Lactuca sativa* L.) with humic substance-based biostimulant applications. Plants.

[63] Álvarez-González A., Serrano L., Gorchs G., Uggetti E. (2025). Exploring the biostimulant potential of *Scenedesmus* sp. grown in wastewater: Impacts on plant growth and photosynthetic activity of lettuce. Chemosphere.

[64] Velasco-Clares D., Navarro-León E., Atero-Calvo S., Ruiz J.M., Blasco B. (2024). Is the application of bioactive anti-stress substances with a seaweed-derived biostimulant effective under adequate growth conditions?. Physiol. Plant..

[65] Cao H., Wang J., Dong X., Han Y., Ma Q., Ding Y., Zhao F., Zhang J., Chen H., Xu Q. (2015). Carotenoid accumulation affects redox status, starch metabolism, and flavonoid/anthocyanin accumulation in citrus. BMC Plant Biol..

[66] Li Y., Jian Y., Mao Y., Meng F., Shao Z., Wang T., Zheng J., Wang Q., Liu L. (2022). “Omics” insights into plastid behavior toward improved carotenoid accumulation. Front. Plant Sci..

[67] Arslan Y., Köklü Ş., Yakupoğlu G. (2022). The effect of melatonin treatments on cauliflower and broccoli seedlings on salt stress. Harran Tarım ve Gıda Bil. Dergisi.

[68] Agati G., Azzarello E., Pollastri S., Tattini M. (2012). Flavonoids as antioxidants in plants: Location and functional significance. Plant Sci..

[69] Rouphael Y., Carillo P., Colla G., Fiorentino N., Sabatino L., El-Nakhel C., Giordano M., Pannico A., Cirillo V., Shabani E. (2020). Appraisal of combined applications of Trichoderma virens and a biopolymer-based biostimulant on lettuce agronomical, physiological, and qualitative properties under variable N regimes. Agronomy.

[70] Živanović B., Milić Komić S., Tosti T., Vidović M., Prokić L., Veljović Jovanović S. (2020). Leaf soluble sugars and free amino acids as important components of abscisic acid–mediated drought response in tomato. Plants.

[71] Jalal A., de Oliveira Junior J.C., Ribeiro J.S., Fernandes G.C., Mariano G.G., Trindade V.D.R., Reis A.R.D. (2021). Hormesis in plants: Physiological and biochemical responses. Ecotoxicol. Environ. Saf..

[72] Erofeeva E.A. (2022). Hormesis in plants: Its common occurrence across stresses. Curr. Opin. Toxicol..

